# Identification of essential oils with strong activity against stationary phase *Mycobacterium abscessus*

**DOI:** 10.1016/j.heliyon.2024.e27073

**Published:** 2024-02-24

**Authors:** Dan Cao, Xiuzhi Jiang, Tiantian Wu, Yanghui Xiang, Jiaying Liu, Zhen Li, Xin Yuan, Kefan Bi, Xu Dong, Tone Tønjum, Kaijin Xu, Ying Zhang

**Affiliations:** a State Key Laboratory for Diagnosis and Treatment of Infectious Diseases, National Clinical Research Center for Infectious Diseases, Collaborative Innovation Center for Diagnosis and Treatment of Infectious Diseases, The First Affiliated Hospital, Zhejiang University School of Medicine, Hangzhou, China; b Department of Microbiology, University of Oslo, NO-0372, Oslo, Norway; c Department of Microbiology, Oslo University Hospital, NO-0424, Oslo, Norway; d Jinan Microecological Biomedicine Shandong Laboratory, Jinan, 250117, China

**Keywords:** *Mycobacterium abscessus*, Stationary phase, Essential oils, Antimicrobial activity, Persisters

## Abstract

**Purpose:**

To identify essential oils (EOs) active against non-growing stationary phase *Mycobacterium abscessus* and multidrug-resistant *M. abscessus* strains.

**Methods:**

The activity of EOs against both stationary and log phase *M. abscessus* was evaluated by colony forming unit (CFU) assay and minimum inhibitory concentration (MIC) testing.

**Results:**

We assessed the activity of 80 EOs against stationary phase *M. abscessus* and found 12 EOs (Cinnamon, Satureja montana, Palmarosa, Lemon eucalyptus, Honey myrtle, Combava, Health shield, Mandarin, Thyme, Rosewood, Valerian Root and Basil) at 0.5% concentration to be active against both growing and non-growing stationary phase *M. abscessus.* Among them, Satureja montana essential oil and Palmarosa essential oil could eliminate all stationary phase *M. abscessus* at 0.125% and Cinnamon essential oil could eliminate stationary phase bacteria at 0.063% after 1-day treatment. Interestingly, these EOs also exhibited promising activity against multidrug-resistant *M. abscessus* clinical strains.

**Conclusions:**

Our study indicates that some EOs display outstanding effectiveness against both drug susceptible *M. abscessus* and multidrug-resistant *M. abscessus* isolates. These findings may be significant for the treatment of persistent *M. abscessus* infections.

## 1 Introduction

*Mycobacterium abscessus* is a fast-growing nontuberculous mesophilic mycobacterium frequently found in water and soil [1]. It is a newly emerged opportunistic pathogen that can lead to severe pulmonary, soft issue and skin diseases, bacteremia and other infections [2]. *M. abscessus* can cause difficult-to-cure lung infections in diseases such as cystic fibrosis or structural lung disorders including COPD which require long-term multidrug regimens because of complex intrinsic and acquired resistance of the organism, as well as lack of bactericidal activity of most antibiotics against this organism. In addition, *M. abscessus* is an increasingly recognized cause of soft tissue and skin infection, both in the hospital and community settings, affecting both immunocompromised and immunocompetent individuals [3]. There is presently no official standardized regimen for treating *M. abscessus* lung infections [4]. Commonly used antibiotics for *M. abscessus* infections include clarithromycin, imipenem, amikacin, azithromycin, tigecycline, clofazimine, linezolid, moxifloxacin, etc. The combination of antibiotics used to treat *M. abscessus* infections depends on the macrolide sensitivity and amikacin sensitivity of the clinical isolates [4]. However, clinical practice has found that there is a lack of correlation between the minimum inhibitory concentration (MIC) of antimycobacterial agents measured *in vitro* and clinical treatment efficacy [5]. The importance of persisters (enriched in stationary phase bacteria) in causing severe persistent infections has recently been demonstrated in *Borrelia burgdorferi, Staphylococcus aureus* and *Pseudomonas aeruginosa* infections [[6], [7], [8]]. Studies have found the existence of *M. abscessus* persisters that are non-replicating and display tolerance to commonly used antibiotics, which may hinder the clearance of NTM infections in patients [9]. Therefore, identification of new agents active against *M. abscessus* persisters would be of interest to develop more effective treatments for this pathogen.

Essential oil (EO) is a hydrophobic fluid extracted from selected parts of plants. Due to their antibacterial, antifungal, antioxidant, antiseptic, and anti-inflammatory properties, EOs are extensively utilized in food processing and pharmaceutical treatment [10]. Many studies have revealed that EOs exhibit antibacterial effects against both Gram-negative and Gram-positive bacteria [11,12]. Moreover, various active ingredients in EOs, including cinnamaldehyde and carvacrol, have shown effectiveness against diverse bacterial strains [13]. However, there is limited research on the antibacterial effects of EOs against *M. abscessus*, with only ginger and Valencia orange oil so far being shown to be active against this organism [14,15]. The purpose of this study is to assess a large collection of EOs and identify promising EOs that demonstrate efficacy against *M. abscessus*.

## 2 Materials and methods

### 2.1 Bacterial strain and culture conditions

*Mycobacterium abscessus* type strain ATCC19977 was acquired from ATCC. Clinical isolates 49, 97, 2136 and 2338 of *M. abscessus* were acquired from The First Affiliated Hospital of Zhejiang University. A single colony from a 7H11 agar plate was inoculated into a 50 mL centrifuge tube containing 10 mL 7H9 broth with 0.2% glycerol and 10% OADC and shaken (220 rpm) at 37 °C. After reaching an OD value of 0.6–0.8, a 1:100 subculture was initiated in 50 mL centrifuge tube. A 5-day stationary phase culture (about 3 × 10^9^/mL) was used for screening essential oils for antibacterial study. For MIC testing, bacterial suspension was standardized to McFarland turbidity of 0.5 and diluted 200-fold in CAMHB medium, resulting in a bacterial count of about 5 × 10^5^/mL.

### 2.2 Antibiotics and essential oils

Amikacin, moxifloxacin, clarithromycin, tigecycline, azithromycin, rifabutin, meropenem, cefoxitin, ciprofloxacin and linezolid were purchased from Macklin (Shanghai, China). Water-soluble antibiotics such as amikacin, meropenem, and cefoxitin were dissolved in ddH2O, while other antibiotics, which are less soluble in water, were dissolved in DMSO. Cinnamaldehyde and carvacrol were obtained from Acmec (Shanghai, China). A collection of 80 commercially available EOs (Table S1) were purchased from Ningbo Ge Blueprint Electronic Industry and Trade Co., Ltd (Ningbo, China), Poli Aromatic Pharmaceutical Technology Co., Ltd (Shanghai, China), Guangzhou La Gu Na Biotechnology Co., Ltd (Guangzhou, China), Prime Time Commerce (Scottsdale Arizona, USA), Healing solutions (USA), Fabulous Frannie (USA) and Plant Therapy (USA). EOs and the EO active components Cinnamaldehyde and Carvacrol were dissolved in DMSO at 20% (v/v) and then diluted using stationary phase or log phase cultures to evaluate their activity against *M. abscessus* in MIC and drug exposure tests (see below). When the concentration of the EO is 0.25%, the concentration of DMSO is 1%.

### 2.3 Evaluation of essential oils for efficacy on stationary phase *M. abscessus*

To assess the activity of EOs against stationary phase *M. abscessus*, EOs and antibiotics were included into 96-well plates with stationary phase *M. abscessus* ATCC19977. Each essential oil was tested at the concentrations of 0.5%, 0.25%, and 0.125% (v/v). Moxifloxacin and tigecycline were utilized at 50 μM as control drugs. The 96-well plates were kept stationary at 37 °C in the incubator. To assess bacterial survival, the bacteria exposed to EOs were transferred to 7H11 plates using a 96-pin replicator after one day or three days according to growth (inactive) or no growth (active) on 7H11 agar plates as previously described [12]. Briefly, a 96-pin replicator was placed into the 96-well plate, with each pin corresponding to a well. Then, we used the 96-pin replicator to evenly mix the liquid in each well of the 96-well plate and then transferred the replicator to a 15 cm 7H11 agar plate to monitor bacterial growth or inhibition. After 4 days, the bacterial growth on the 7H11 agar plates was observed. If no growth was observed at a certain position on the plates, it is considered that the essential oil in the corresponding well is active.

### 2.4 Antimicrobial susceptibility test

The minimum inhibitory concentrations (MICs) against *M. abscessus* 19977 and four *M. abscessus* clinical isolates including *M. abscessus* 49, 97, 2136 and 2338 were determined using the microdilution method following the Clinical and Laboratory Standards Institute (CLSI) guidelines [16]. In short, a 2-fold dilution of EOs from 1% to 0.016% was performed. Amikacin, moxifloxacin, clarithromycin, meropenem, cefoxitin, ciprofloxacin and linezolid were diluted twofold from 128 μg/mL to 0.063 μg/mL. The 96-well plates were incubated 3–4 days at 37 °C to assess visible inhibition of bacterial growth.

### 2.5 Confirmation of active essential oil activity by CFU count

The active EOs identified in the preliminary screening were further tested in drug exposure by CFU assay. Stationary phase bacterial cultures and an appropriate amount of 20% EO were added to Eppendorf tubes to achieve the desired concentration of 0.5%, 0.25% and 0.125%, respectively. Amikacin, moxifloxacin, clarithromycin, tigecycline, azithromycin, rifabutin, and linezolid were included in the bacterial suspension at 50 μM. Stationary phase *M. abscessus* culture without drugs or EOs was used as drug-free control. The drug-free control sample was prepared by diluting stationary bacteria in a mixed solution of 80% DMSO and 20% PBS (4:1 ratio), the same way as the dilution of EOs. For example, 0.25% EO sample contains 1% DMSO. Our experimental observations revealed that the 4% DMSO had no effect on stationary phase *M. abscessus* survival compared to PBS. At 1, 3, 5, 7 days, the bacterial suspensions were washed, resuspended in PBS, serially diluted and plated on 7H11 plates. After 3–4 days of incubation at 37 °C, colony forming units (CFUs) were counted. The CFU data of *M. abscessus* treated with EOs were presented using mean ± standard deviation in figures and tables.

## 3 Results

### 3.1 Screening of essential oils for activity against stationary phase *M. abscessus*

Stationary phase bacteria enriched with non-growing persisters were previously used as a model for high-throughput drug screening against persisters for various bacterial pathogens such as uropathogenic *Escherichia coli* and *Borrelia burgdorgeri* [11,17]. Here we assessed a collection of 80 EOs against stationary phase *M. abscessus*. After 1-day treatment, eleven (Cinnamon, Satureja montana, Palmarosa, Lemon eucalyptus, Honey Myrtle, Combava, Health shield, Mandarin, Thyme, Rosewood, Valerian Root), five (Cinnamon, Satureja montana, Palmarosa, Health shield, Mandarin) and two (Cinnamon and Satureja montana) EOs were found to exhibit high activity against *M. abscessus* at 0.5, 0.25 and 0.125%, respectively. After 3-day treatment, an additional essential oil (Basil) demonstrated activity at 0.5% (Table 1). In contrast, moxifloxacin and tigecycline at 50 μM were unable to kill all stationary phase *M. abscessus* even after 7-day treatment.

**Table 1:** Effect of essential oils (EOs) on stationary phase *M. abscessus*.

EO	Viability of bacteria after 1 or 3 days of exposure					
	0.5%EO		0.25%EO		0.125%EO	
	1day	3day	1day	3day	1day	3day
Cinnamon	–	–	–	–	–	–
Satureja montana	–	–	–	–	–	–
Health shield	–	–	–	–	+	+
Mandarin	–	–	–	–	+	+
Palmarosa	–	–	–	–	+	+
Lemon eucalyptus	–	–	+	+	+	+
Honey myrtle	–	–	+	+	+	+
Combava	–	–	+	+	+	+
Thyme	–	–	+	+	+	+
Rosewood	–	–	+	+	+	+
Valerian root	–	–	+	+	+	+
Basil	+	–	+	+	+	+

The stationary phase *M. abscessus* was incubated with EOs or control drugs moxifloxacin and tigecycline (both at 50 μM) in 96-well microtiter plates at 37 °C for 1 day or 3 days when the bacterial survival was examined by transferring the bacterial suspension to 7H11 agar plates with a 96-pin replicator to monitor any bacterial growth after incubation for 5 days. “-” indicates no obvious growth on 7H11 plates after exposure; “+“ indicates obvious growth on 7H11 plates after exposure.

### 3.2 MICs of the active essential oils for *M. abscessus*

Drug susceptibility testing was conducted to evaluate the effectiveness of active EOs from the above screen for growing *M. abscessus* including *M. abscessus* 19977 and four multidrug-resistant clinical isolates which are especially resistant to clarithromycin including *M. abscessus* 49, 97, 2136 and 2338. The drug susceptibility result of clinical strains is displayed in Table S2. As shown in Table 2, Cinnamon essential oil displayed the most potent activity in hindering the growth of *M. abscessus* with the MIC of 0.032%. The growth of *M. abscessus* was inhibited by Satureja montana essential oil, with an MIC ranging from 0.063% to 0.125%. Health shield and Mandarin essential oils were able to suppress the growth of *M. abscessus* at 0.125%. The growth of *M. abscessus* was inhibited by Palmarosa, Thyme, Valerian Root and Basil essential oils from 0.125% to 0.25%. In addition, Honey myrtle, Lemon eucalyptus and Rosewood essential oils also showed antibacterial effects against *M. abscessus*.

**Table 2:** Minimum inhibitory concentrations (MICs, expressed in v/v %) of active essential oils against *M. abscessus* 19977 and four multidrug-reisistant clinical isolates.

Isolate	19977	49	97	2338	2136
Cinnamon	0.032	0.032	0.032	0.032	0.032
Satureja montana	0.125	0.063	0.063	0.125	0.125
Palmarosa	0.125	0.125	0.125	0.25	0.125
Health shield	0.125	0.125	0.125	0.125	0.125
Mandarin	0.125	0.125	0.125	0.125	0.125
Honey myrtle	0.25	0.25	0.25	0.25	0.25
Combava	0.25	0.5	0.5	0.5	0.5
Thyme	0.25	0.25	0.125	0.125	0.125
Valerian Root	0.25	0.25	0.25	0.125	0.125
Basil	0.25	0.25	0.25	0.125	0.25
Lemon eucalyptus	0.5	0.5	0.5	0.5	0.5
Rosewood	0.5	1	1	1	1

### 3.3 Comparison of active essential oils and commonly used drugs for their abilities to kill stationary phase *M. abscessus*

**Fig. 1:**
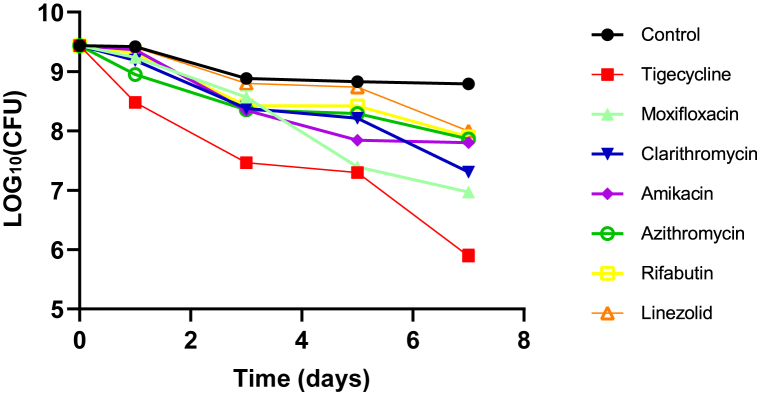
**Activity of commonly utilized drugs against stationary phase *M. abscessus*.** Stationary phase *M. abscessus* was exposed to the antibiotics used to treat *M. abscessus* infections for 1, 3, 5, and 7 days when the CFU was determined. The final concentration of the drugs including amikacin, moxifloxacin, clarithromycin, tigecycline, azithromycin, rifabutin, and linezolid was all 50 μM.

**Fig. 2:**
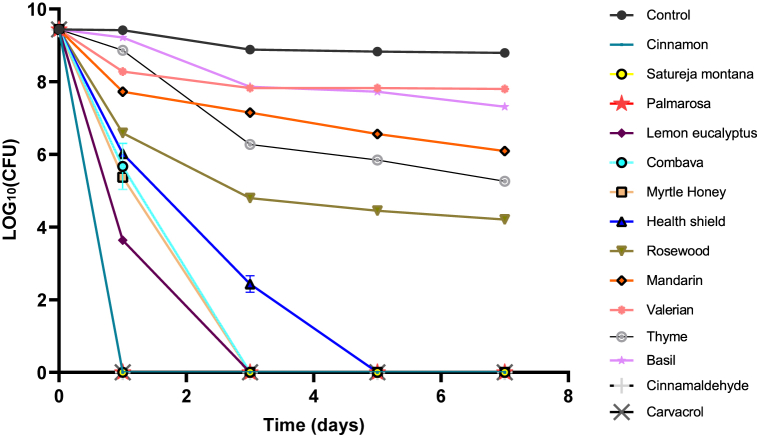
**Activity of active essential oils (0.125%) or their active ingredients (0.063%) against stationary phase *M. abscessus*.** Stationary phase *M. abscessus* was exposed to the active EOs for 1, 3, 5, and 7 days when the CFU was determined. Cinnamon, Satureja montana and Palmarosa could eliminate stationary phase *M. abscessus* after 1-day treatment. Lemon eucalyptus, Honey myrtle and Combava could eliminate bacteria after 3-day treatment. Health shield could kill all *M. abscessus* after 5-day treatment.

In this study, we first tested amikacin, moxifloxacin, clarithromycin, tigecycline, azithromycin, rifabutin, and linezolid for activity against stationary phase *M. abscessus* at 50 μM. However, these commonly used drugs for *M. abscessus* infections showed poor or no obvious activity against non-replicating *M. abscessus* (Fig. 1). In contrast, at 0.5% concentration, 12 EOs (Cinnamon, Satureja montana, Palmarosa, Lemon eucalyptus, Honey myrtle, Combava, Health shield, Mandarin, Thyme, Rosewood, Valerian Root and Basil) were found to kill all *M. abscessus* in 3-day treatment. At 0.25%, eight essential oils (Cinnamon, Satureja montana, Palmarosa, Lemon eucalyptus, Honey myrtle, Combava, Health shield, and Mandarin) were able to eradicate bacteria after 1-day treatment (Table 3). Rosewood essential oil could eliminate all bacteria after 3-day treatment. Thyme essential oil and Valerian Root essential oil could kill all *M. abscessus* after 5 days. At a lower 0.125% concentration, Cinnamon, Satureja montana and Palmarosa essential oils could clear all *M. abscessus* after 1-day treatment (Fig. 2). Lemon eucalyptus, Honey myrtle and Combava essential oils could eliminate all *M. abscessus* after 3-day treatment. Health shield essential oil was able to kill *M. abscessus* with no detectable CFU after 5-day treatment. In addition, Cinnamon essential oil could eliminate *M. abscessus* after 1-day treatment at 0.063%. In contrast, Mandarin, Thyme, Rosewood, Valerian Root and Basil essential oils at 0.125% were not able to eliminate *M. abscessus*, even after 7-day treatment.

**Table 3:** Stationary phase *M. abscessus* culture was treated with essential oils at 0.25% (v/v) at different times followed by CFU count.

Essential oils	CFU/mL after drug exposure			
	1 Day	3 Day	5 Day	7 Day
Cinnamon	0	0	0	0
Satureja montana	0	0	0	0
Palmarosa	0	0	0	0
Lemon eucalyptus	0	0	0	0
Honey myrtle	0	0	0	0
Combava	0	0	0	0
Health shield	0	0	0	0
Mandarin	0	0	0	0
Rosewood	2.27 ± 0.45 × 10^3^	0	0	0
Valerian Root	4.97 ± 0.31 × 10^7^	1.77 ± 0.25 × 10^4^	0	0
Thyme	1.30 ± 0.43 × 10^7^	1.77 ± 0.30 × 10^3^	0	0
Basil	1.20 ± 0.10 × 10^8^	9.00 ± 0.10 × 10^7^	1.50 ± 0.30 × 10^7^	6.33 ± 1.52 × 10^6^
Control	2.63 ± 0.15 × 10^9^	7.67 ± 0.65 × 10^8^	6.77 ± 0.25 × 10^8^	6.2 ± 0.26 × 10^8^

### 3.4 Cinnamaldehyde and carvacrol as highly potent active essential oil ingredients against stationary phase *M. abscessus*

Cinnamaldehyde and carvacrol are known active components in cinnamon bark and Satureja montana, oregano or thyme essential oils, respectively [13,18]. We tested cinnamaldehyde and carvacrol for their antimicrobial activity against *M. abscessus* (Fig. 2). Cinnamaldehyde was active with the MIC of 0.032% and rapidly eliminated stationary phase *M. abscessus* with no detectable CFU after 1-day treatment at a low concentration of 0.063% in a time-kill drug exposure experiment. In addition, we found that the growth of *M. abscessus* was effectively inhibited by carvacrol at 0.063%. Carvacrol was able to eliminate all stationary phase *M. abscessus* after 1-day treatment at 0.063%. Since the killing curves for cinnamaldehyde and carvacrol in Fig. 2 overlapped with those of Cinnamon, Satureja montana and Palmarosa EO treatment, the detailed CFU information is shown in Table S3 to present the results more clearly.

## 4 Discussion

*M. abscessus* is an opportunistic multidrug-resistant NTM which causes notoriously persistent lung infections, as well as infections in many other body sites that are difficult to cure, especially in patients with cystic fibrosis [19]. Despite long-term treatment with multiple antibacterial drugs, the pooled sputum culture conversion rate in patients with *M. abscessus* infection was only about 40% [20]. An important reason for the failure of treatment may be the lack of direct correlation between the MIC of antibiotics measured and the actual clinical outcome [5]. Normally, the MIC of an antimicrobial is the concentration that prevents the growth of a planktonic culture cultivated in aerated, nutrient-rich broth. However, the microenvironment of M. abscessus inside the macrophage in the host may include hypoxia, nutrient limitation and oxidative and nitrosative stress [[21], [22], [23], [24]], which may facilitate the development of slowly growing or non-growing persisters, that are tolerant to current antibiotics [25]. It is worth noting that drugs such as amikacin, clarithromycin, and moxifloxacin, which are currently used for the treatment of *M. abscessus* infections, are effective against growing bacteria, but exhibit limited activity against non-replicating persisters. Therefore, there is an urgent need and significant interest to discover drugs with bactericidal activity against non-growing persisters, including *M. abscessus* persisters [26,27], which are enriched in stationary phase bacteria. There has been limited studies on the antimicrobial effects of EOs against *M. abscessus*. One previous study discovered that the MIC of ginger EO against *M. abscessus* was 125 μg/ml [14]. In addition, Valencia orange oil was found to have activity against *M. avium* complex and *M. abscessus* [15]. Here, we evaluated a large collection of 80 EOs and identified 12 EOs (Cinnamon, Satureja montana, Palmarosa, Lemon eucalyptus, Honey myrtle, Combava, Health shield, Mandarin, Thyme, Rosewood, Valerian Root and Basil) that are highly active against both log phase *M. abscessus* and non-replicating stationary phase *M. abscessus*. In particular, these essential oils also demonstrated promising antimicrobial activity against multidrug-resistant clinical isolates especially clarithromycin-resistant clinical strains.

Essential oils have been utilized for centuries as a natural remedy to combat various infections [28]. Studies have revealed that some EOs demonstrate remarkable bactericidal activity against stationary phase *Borrelia burgdorferi,* uropathogenic *Escherichia coli, Staphylococcus aureus* and *Bartonella henselae* [[11], [12], [13],^21^]. In our study, Cinnamon EO extracted from Cinnamomum zeylanicum exhibited the most potent activity against *M. abscessus*. Cinnamon is extensively utilized for its antiseptic, antioxidant, and antibacterial properties. The presence of cinnamaldehyde and eugenol in cinnamon oil accounts for its significant antimicrobial activity [29]. In previous studies, we found Cinnamon EO could rapidly kill various pathogens [[11], [12], [13],^21^]. Therefore, we tested the antibacterial and bactericidal activities of Cinnamon EO and its active ingredient cinnamaldehyde. Here, the growth of *M. abscessus* was efficiently suppressed by cinnamon and cinnamaldehyde at a very low concentration of 0.032%. And cinnamon and cinnamaldehyde both had remarkable anti-persister activity and could clear all *M. abscessus* persisters after 1-day treatment at a low concentration (0.063%).

Additionally, we found that Satureja montana, Palmarosa, Lemon eucalyptus, Combava, Honey myrtle and Health shield EOs showed remarkable activity against stationary phase *M. abscessus*. At 0.125%, these EOs exhibited significant efficacy against stationary phase *M. abscessus* and eliminated all bacteria in 7-day exposure (Fig. 2). Actually, they have been reported to display excellent antimicrobial properties. The Satureja montana extracts were effective against a wide variety of bacterial species, including *Staphylococcus aureus*, *Streptococcus dysgalactiae*, *Klebsiella pneumoniae* and *Pseudomonas aeruginosa* [30]. Carvacrol is the active compound in Satureja montana EO [18] and showed excellent antibacterial activity in our study. Eucalyptus essential oil has been traditionally used for a variety of diseases, including tuberculosis, fungal infections, and influenza, based on the antimicrobial capacity of its EO [31]. In our previous study, Palmarosa essential oil showed high activity against non-growing *S. aureus* at 0.125% [12]. Additionally, Palmarosa oil was investigated as an antimicrobial agent for reducing antibiotic resistance and natural preservatives to treat skin infections [32,33] and geraniol was identified as a major active component of Palmarosa essential oil [34]. However, further studies are necessary to validate if geraniol is the active ingredient against *M. abscessus. Combava* essential oil*,* also known as *Citrus hystrix*, exhibited antibacterial activity against *Haemophilus* influenzae*,* Streptococcus pneumoniae and *Staphylococcus* aureus [35]. Acacia honey and myrtle extracts have the capability to inhibit the cariogenic bacteria [36]. Health Shield essential oil is a synergistic essential oil blend that includes cassia, clove, eucalyptus, lemon, and rosemary essential oils. In our previous study, Health Shield essential oil could clear all uropathogenic *Escherichia coli* persisters at 1% after 1-day exposure [11]. Therefore, the high activity of the above EOs against *M. abscessus* may provide evidence to expand usage against mycobacterial infections. Further studies to discover the bioactive ingredients and the mechanisms of action against *M. abscessus* persisters are warranted.

Some other EOs identified to be effective against *M. abscessus* in our research have also been proved to have significant activity against other bacteria in previous studies. Mandarin essential oil has been proven to possess antimicrobial activity against a range of pathogens including *Staphylococcus aureus* and *Aeromonas hydrophila* [37,38]. Rosewood EO has been extensively utilized in the perfumery industry [39]. We previously found rosewood to be active against *Staphylococcus aureus* perisisters [12]. And thyme essential oil has been utilized in the treatment of parasitic infections, coughs and upper respiratory tract infections [40]. It demonstrates antibacterial effects against both Gram-negative and Gram-positive bacteria and exhibits antiviral, antifungal, antioxidant and anti-inflammatory properties [40], and its active ingredients include thymol and carvacrol.

There were some seemingly contradictory results between the initial screening and CFU validation in our study. There was visible growth on 7H11 plates after transfer from 96-well plate with Lemon eucalyptus, Honey myrtle, and Combava EOs at 0.25% in Table 1, but no CFU were counted in validation experiment in Eppendorf tubes as shown in Table 3. This is probably because our initial screening experiments was conducted in a 96-well plate (Table 1), which may have caused evaporation of EOs, while the CFU validation (Table 3) was performed in Eppendorf tubes with closed lid that prevented evaporation of EOs. Given the inherent volatility of essential oils, and the non-sealed nature of the 96-well plate, it is plausible that some degree of oil evaporation occurred, which potentially affected its antibacterial efficacy.

Essential oils have significant cytotoxic properties that may affect their use in vivo. However, despite the cytotoxic limitations associated with essential oils, it is important to note that some EOs have been shown to have the potential to treat skin infections. Various formulations, such as gel, or ointment bases, were developed to assess the efficacy of essential oils as antifungal agents in skin infection model [41]. It has been found that eucalyptus oil loaded in lipid nanoparticles promotes wound healing in vivo, which demonstrates the potential of essential oils in treating skin infections [42]. Further studies with the active EOs identified in this study in *M. abscessus* skin infection model are warranted.

## 5 Conclusion

To summarize, this study is the first to screen a large panel of 80 EOs for their potential activity against both growing and stationary phase *M. abscessus*. The following promising EOs, including Cinnamon, Satureja montana, Palmarosa, Lemon eucalyptus, Honey myrtle, Combava, Health shield, Mandarin, Thyme, Rosewood, Valerian Root, and Basil, have been identified as highly active against *M. abscessus*. Further research is required to identify the distinctly active compounds and their antimicrobial mode of action, as well as to evaluate the safety and efficacy of the promising EOs in the mouse model of infection in the near future.

## Additional information

None.

## Data availability statement

Data included in this study are available upon request.

## CRediT authorship contribution statement

**Dan Cao:** Writing – original draft, Validation, Methodology. **Xiuzhi Jiang:** Methodology. **Tiantian Wu:** Formal analysis. **Yanghui Xiang:** Methodology. **Jiaying Liu:** Methodology. **Zhen Li:** Validation. **Xin Yuan:** Validation. **Kefan Bi:** Writing – original draft. **Xu Dong:** Formal analysis. **Tone Tønjum:** Writing – review & editing. **Kaijin Xu:** Writing – review & editing. **Ying Zhang:** Writing – review & editing, Funding acquisition, Conceptualization.

## Declaration of competing interest

The authors declare that they have no known competing financial interests or personal relationships that could have appeared to influence the work reported in this paper.
