# Supplementary material for: Identification of essential oils with strong activity against stationary phase Mycobacterium abscessus
Source: Heliyon. 2024 Feb 24;10(5):e27073. doi: 10.1016/j.heliyon.2024.e27073 (PMC10920374; doi:10.1016/j.heliyon.2024.e27073)
Supplement: Multimedia component 1 [file mmc1.docx]

[**Table S1**](https://www.ncbi.nlm.nih.gov/pmc/articles/PMC7092464/bin/12906_2020_2898_MOESM1_ESM.docx)**. Antibacterial activity of 80 EOs evaluated against stationary phase *M. abscessus***

| **Essential oil** | **Antibacterial activity^a^** |
| --- | --- |
| 1. Basil | active |
| 1. Cinnamon | active |
| 1. Combava | active |
| 1. Health shield | active |
| 1. Honey myrtle | active |
| 1. Lemon eucalyptus | active |
| 1. Mandarin | active |
| 1. Palmarosa | active |
| 1. Rosewood | active |
| 1. Satureja montana | active |
| 1. Thyme | active |
| 1. Valerian Root | active |
| 1. Artemisia | inactive |
| 1. Bay Sweet Tree | inactive |
| 1. Berganot | inactive |
| 1. Black Seed | inactive |
| 1. Breathe Blend | inactive |
| 1. Cajeput | inactive |
| 1. Camphor | inactive |
| 1. Carrot Seed | inactive |
| 1. Cedar Wood | inactive |
| 1. Chamomile | inactive |
| 1. Clary Sage | inactive |
| 1. Copaiba Balsam | inactive |
| 1. Cornmint | inactive |
| 1. Coriander | inactive |
| 1. Cryptolepis | inactive |
| 1. Curcuma | inactive |
| 1. Cypress | inactive |
| 1. Dill Herb | inactive |
| 1. Eucalyptus | inactive |
| 1. Fennel | inactive |
| 1. Galbanum | inactive |
| 1. Grapefruit | inactive |
| 1. Head Ease | inactive |
| 1. Hu Zhang | inactive |
| 1. Huang Qin | inactive |
| 1. Hyssop | inactive |
| 1. Juniper Berry | inactive |
| 1. Kannka | inactive |
| 1. Lantana | inactive |
| 1. Laste. Tree | inactive |
| 1. Lavender | inactive |
| 1. Lemon | inactive |
| 1. Lime | inactive |
| 1. Marjoram | inactive |
| 1. Melissa | inactive |
| 1. Michelia Alba Flower | inactive |
| 1. Neem Oil | inactive |
| 1. Neroil | inactive |
| 1. Niaouli | inactive |
| 1. Nutmeg | inactive |
| 1. Peppermint· | inactive |
| 1. Petitgrain | inactive |
| 1. Pine | inactive |
| 1. Pine Sylvestre | inactive |
| 1. Pine Tree | inactive |
| 1. Ravensara | inactive |
| 1. Ravintsara | inactive |
| 1. Rhododendron | inactive |
| 1. Rose | inactive |
| 1. Rosemary | inactive |
| 1. Siberian Fir | inactive |
| 1. Silver Fir | inactive |
| 1. Spearmint | inactive |
| 1. Spruce Black | inactive |
| 1. Sweet Fennel | inactive |
| 1. Sweet. Orange | inactive |
| 1. Tangerine | inactive |
| 1. Tarragon | inactive |
| 1. Tea Tree | inactive |
| 1. Trankincense | inactive |
| 1. Uncaria Sinensis | inactive |
| 1. Verbena Spanish | inactive |
| 1. Vetiver | inactive |
| 1. Wintergreen | inactive |
| 1. Wormwood | inactive |
| 1. Yarrow Chamazulene | inactive |
| 1. Ylang Ylang | inactive |
| 1. Zanthoxylum | inactive |

^a^ Essential oils (EOs) at 0.5% were initially screened for their antibacterial activity against *M. abscessus* after 3-days exposure by a 96-pin replicator to monitor bacterial survival according to growth (inactive) or no growth (active) on 7H11 plates.

**Table S2. Antimicrobial agents and drug susceptibility of clinical isolates of** ***M. abscessus^a^***

| **Isolate** | **Amikacin** | **Moxifloxacin** | **Clarithromycin** | **Meropenem** | **Cefoxitin** | **Ciprofloxacin** | **Linezolid** |
| --- | --- | --- | --- | --- | --- | --- | --- |
| 49 | Susceptible | Resistant | Resistant | Intermediate | Intermediate | Resistant | Intermediate |
| 97 | Susceptible | Resistant | Resistant | Intermediate | Intermediate | Resistant | Susceptible |
| 2136 | Susceptible | Resistant | Resistant | Resistant | Intermediate | Resistant | Intermediate |
| 2338 | Susceptible | Resistant | Resistant | Resistant | Resistant | Resistant | Intermediate |

^a^This test was performed according to CLSI standards (document M62) for susceptibility testing of mycobacteria. For details, see Methods section.

**Table S3. Stationary phase *M. abscessus* culture was treated with active essential oils (0.125%) or their active ingredients (0.063%) at different times followed by CFU count.**

| **Essential oils** | **CFU/mL after drug exposure** | | | |
| --- | --- | --- | --- | --- |
|  | **1 Day** | **3 Day** | **5 Day** | **7 Day** |
| Control | 2.63±0.15× 10^9^ | 7.67±0.65× 10^8^ | 6.77±0.25× 10^8^ | 6.2±0.26× 10^8^ |
| Cinnamon | 0 | 0 | 0 | 0 |
| Satureja montana | 0 | 0 | 0 | 0 |
| Palmarosa | 0 | 0 | 0 | 0 |
| Cinnamaldehyde | 0 | 0 | 0 | 0 |
| Carvacrol | 0 | 0 | 0 | 0 |
| Lemon eucalyptus | 4.33±0.58x 10^3^ | 0 | 0 | 0 |
| Honey myrtle | 2.37±0.32x 10^5^ | 0 | 0 | 0 |
| Combava | 9.70±13.25x 10^5^ | 0 | 0 | 0 |
| Health shield | 1.07±0.23x 10^6^ | 3.00±1.73x 10^2^ | 0 | 0 |
| Mandarin | 5.33±0.58x 10^7^ | 1.43±0.32x 10^7^ | 3.67±0.58x 10^6^ | 1.23±0.12x 10^6^ |
| Rosewood | 3.83±0.15x 10^6^ | 6.33±1.53x 10^4^ | 2.83±0.35x 10^4^ | 1.63±3.79x 10^4^ |
| Valerian Root | 1.90±0.10x 10^8^ | 6.67±0.58x 10^7^ | 6.67±0.58x 10^7^ | 6.33±5.77x 10^7^ |
| Thyme | 7.33±0.58x 10^8^ | 1.90±0.36x 10^6^ | 7.00+1.00x 10^5^ | 1.83±0.21x 10^5^ |
| Basil | 1.67±0.25x 10^9^ | 7.33±1.53x 10^7^ | 5.33±0.58x 10^7^ | 2.07±0.31x 10^7^ |
